# Endothelial glycocalyx in hepatopulmonary syndrome: An indispensable player mediating vascular changes

**DOI:** 10.3389/fimmu.2022.1039618

**Published:** 2022-12-22

**Authors:** Liang Li, Christopher Cook, Yale Liu, Jianzhong Li, Jiantao Jiang, Shaomin Li

**Affiliations:** ^1^ Department of Thoracic Surgery, the Second Affiliated Hospital of Xi’an Jiaotong University, Xi’an, Shaanxi, China; ^2^ Division of Immunology and Pathogenesis, Department of Molecular and Cell Biology, University of California, Berkeley, Berkeley, CA, United States; ^3^ Department of Dermatology, the Second Affiliated Hospital of Xi’an Jiaotong University, Xi’an, Shaanxi, China

**Keywords:** hepatopulmonary syndrome, endothelial glycocalyx, intrapulmonary vascular dilatations, angiogenesis, nitric oxide, endothelial cell, monocyte

## Abstract

Hepatopulmonary syndrome (HPS) is a serious pulmonary vascular complication that causes respiratory insufficiency in patients with chronic liver diseases. HPS is characterized by two central pathogenic features—intrapulmonary vascular dilatation (IPVD) and angiogenesis. Endothelial glycocalyx (eGCX) is a gel-like layer covering the luminal surface of blood vessels which is involved in a variety of physiological and pathophysiological processes including controlling vascular tone and angiogenesis. In terms of lung disorders, it has been well established that eGCX contributes to dysregulated vascular contraction and impaired blood-gas barrier and fluid clearance, and thus might underlie the pathogenesis of HPS. Additionally, pharmacological interventions targeting eGCX are dramatically on the rise. In this review, we aim to elucidate the potential role of eGCX in IPVD and angiogenesis and describe the possible degradation-reconstitution equilibrium of eGCX during HPS through a highlight of recent literature. These studies strongly underscore the therapeutic rationale in targeting eGCX for the treatment of HPS.

## 1 Introduction

Hepatopulmonary syndrome (HPS) is a serious vascular complication that causes respiratory insufficiency in patients with chronic liver diseases. The incidence of HPS ranges from 5 to 32% in the setting of liver cirrhosis and markedly increases the mortality of affected patients (1). HPS develops with two central pathogenic features—pulmonary microvascular dilatation and angiogenesis, which collectively lead to gas exchange abnormality and impaired oxygenation in the absence of intrinsic cardiopulmonary diseases. Abnormal oxygenation can be diagnosed clinically by an elevated alveolar-arterial oxygen gradient while changes in intrapulmonary microvascular dilatation are now routinely assessed through contrast-enhanced transthoracic echocardiography. Despite advancements in diagnosis, liver transplantation remains the only effective therapeutic option for HPS. Investigations based on the animal model of common bile duct ligation (CBDL) have provided significant progresses towards effective HPS therapy (2, 3). However, a huge gulf continues to separate the bench from the bedside, thus necessitating a comprehensive understanding of the mechanisms underlying the pathogenesis of HPS.

The luminal surface of blood vessels is covered by a polysaccharide-abundant gel-like layer called endothelial glycocalyx (eGCX). First discovered in 1966 with the aid of transmission electron microscopy, the eGCX is mainly configured by proteoglycans and glycoproteins anchored to the endothelial cell membrane that serve as a foundation for the rest of the glycocalyx constituents (2). The proteoglycans of eGCX are principally syndecans and glypicans. They often present on the endothelium with glycosaminoglycan chains such as heparan sulfate and chondroitin sulfate (3). The composition and structure of eGCX are in a state of dynamic replenishment and are delicately regulated by enzymatic degradation “shedding”, *de novo* biosynthesis of new molecules, and recruitment of circulating molecules from the blood. In addition, eGCX is heterogeneous across different species, vascular beds, organs and shear stress rates based on varying arrangements of glycosaminoglycan chains and composition (4). The existence of eGCX on the surface of endothelium precludes the direct attachment or adhesion of plasma proteins, molecules, and circulating leukocytes. In that, eGCX is deemed to be a protective barrier by preventing the disordered activation of endothelial cells and the disruption of cellular junctions and the basement membrane. Besides protecting endothelium integrity, the eGCX serves as a versatile regulator in microvessels in a variety of physiological and pathophysiological processes like the shear stress response, vascular contraction, coagulation, inflammation, vascular regeneration, and others (2). It has been established that eGCX is highly useful in diagnosis and treatment of many diseases, especially sepsis, acute respiratory distress syndrome (ARDS), and shock. As dysfunction of the eGCX accompanies many disorders, pharmacological interventions targeting this covering layer are dramatically on the rise in the past decade (5, 6).

It has been established that pulmonary eGCX is frequently dysregulated, leading to impaired blood-gas barrier and fluid clearance in lung diseases, and thus may underlie the pathogenesis of HPS (7, 8). However, the detailed molecular mechanisms are not completely understood, masking the clinical utility of eGCX for HPS treatment. In this narrative review, we summarize the recent findings linking eGCX to the pathogenesis of HPS in order to elucidate the potential therapeutic value of targeting eGCX for HPS treatment.

## 2. The role of eGCX in lung diseases

**Figure 1:**
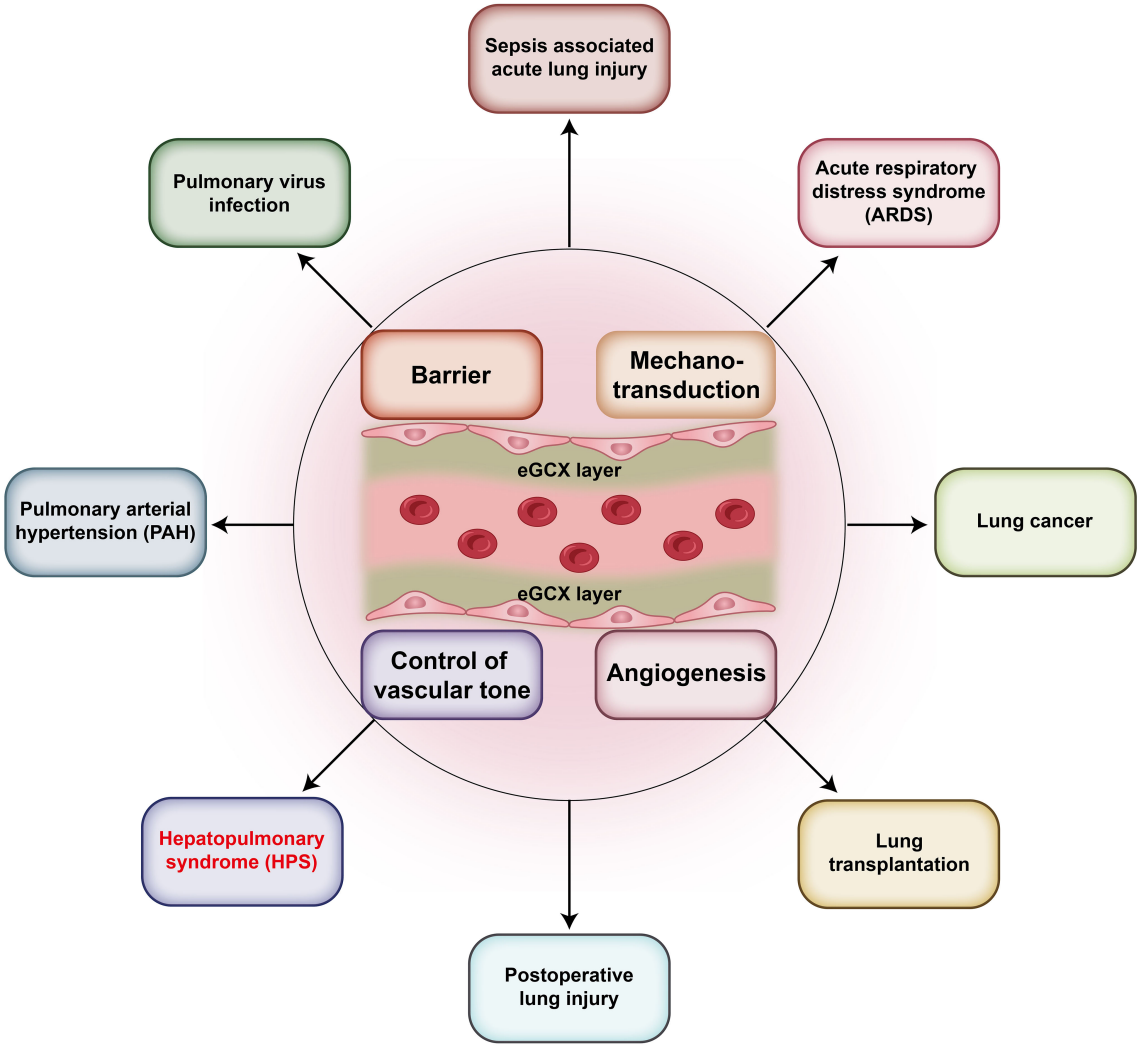
eGCX-related pulmonary diseases. The eGCX has been reported to participate in the pathogenic processes of multiple pulmonary diseases, which include Sepsis associated acute lung injury (ALI), pulmonary virus infection, pulmonary arterial hypertension (PAH), hepatopulmonary syndrome (HPS), postoperative lung injury, lung transplantation, lung cancer, and acute respiratory distress syndrome (ARDS). The mechanisms involve endothelial barrier maintenance, hemodynamic mechanotransduction, vascular tone control, and angiogenesis.

Traditionally, the eGCX was seen as a protective component to keep the integrity of endothelial barrier and defend against circulating insults and stimuli. Recent studies have revealed that the released GCX fragments can act as danger-associated molecular patterns (DAMPs) that activate innate-immune receptors leading to pathogenic consequences (4). Indeed, the roles of eGCX in microcirculation are pleiotropic and multifaceted. The pulmonary blood vessels are one of the most important parts of the microcirculation and are responsible for the collection of almost all the venous blood and circulating antigens. They are the unique constituent of the blood-gas barrier in combination with the alveoli. Therefore, the disturbance of pulmonary eGCX can be more influential and participate in many lung disorders, such as sepsis-associated lung injury and ARDS (9) (**Figure 1**). In fact, it has been found that the pulmonary eGCX layer is thinner than that of other organs which might account for the complex lung defense against internal and external insults (10).

### 2.1 Sepsis associated acute lung injury

Sepsis is a common and severe clinical manifestation characterized by a systemic inflammatory response to infection and has a mortality rate ranging from 17% to 26% (11). To date, it has been acknowledged that sepsis is not merely a simplistic cytokine response but a severe endothelial dysfunction syndrome in response to intravascular and extravascular infection. Interestingly, over 40% of septic patients develop ALI, a syndrome initiated by degradation of the pulmonary eGCX *via* inflammatory mechanisms (12). The inflammatory responses during sepsis are particularly apparent within the pulmonary circulation, which may be correlated to the high-flow and low-pressure blood flow that permits the continuous exposure to primed leukocytes and circulating pathogen/damage associated molecular patterns (PAMPs/DAMPs). The activated leukocytes, especially neutrophils, release reactive oxygen species (ROS) and pro-inflammatory cytokines such as tumor necrosis factor alpha (TNF-α) and interleukin-1β (IL-1β). These cytokines also facilitate the secretion of matrix-degrading enzymes originating from leukocytes themselves, endothelium, or peripheral organs and tissues, and thus contribute to the degradation of eGCX (6, 13). Among these degrading enzymes, a disintegrin and metalloproteinases (ADAMs), heparinase, and hyaluronidase have been demonstrated to play important roles. ADAMs can cleave syndecans, one of the major constituents of eGCX, cytokine receptors, and cell adhesion molecules expressed by endothelial cells and leukocytes (14). As to heparanase, it is capable of degrading heparin sulfate moieties, further aggravating the disruption of eGCX (13, 15). According to the study by Schmidt, et al., eGCX degradation involves the specific loss of heparin sulfate caused by endothelial stored heparanase, a TNF-α–responsive, heparin sulfate–specific glucuronidase (12). The degradation of eGCX increases the exposure of adhesion molecules on endothelium to circulating leukocytes and contributes to neutrophil adhesion. Heparinase inhibition prevented eGCX loss and neutrophil adhesion and, accordingly, attenuated sepsis-induced ALI and mortality in mice. Hyaluronidase cleaves hyaluronic acid (HA) and attenuates the thickness of eGCX. Exogenous administration of high-molecular-weight HA improves sepsis-induced lung injury. In septic lung, eGCX degradation leads to vascular hyper-permeability, abnormal vasodilation, microvascular thrombosis, and augmented leukocyte adhesion, altogether underlying the pathogenesis of ALI (12).

### 2.2 Postoperative lung injury

Postoperative lung injury is the leading cause of death following thoracic surgery. It is mainly related to the procedure of one-lung ventilation (OLV) during the thoracic operation regardless of lung resection (16). Fluid overload, ischemia-reperfusion, and massive transfusion also result in lung damage (17). The eGCX may represent a common pathway for pulmonary injury occurrence because it is affected by most of the aforementioned procedures. During OLV, the eGCX layer can be disrupted by mechanical and nonmechanical stimuli. OLV-associated regional lung overdistension and positive end expiratory pressure (PEEP) can narrow down the pulmonary vascular bed, resulting in the direct attachment of circulating leukocytes and platelets to eGCX (18). As in sepsis, leukocytes produce and/or activate enzymes through secreted proinflammatory cytokines to degrade the eGCX barrier and result in endothelial damage, increased vascular permeability and inflammatory tone, subsequently leading to alveolar injury (19). Platelets with shedded eGCX fractions and damaged vascular endothelium give rise to thrombosis in the pulmonary microcirculation, which progressively exacerbate the ventilation-perfusion mismatch during OLV (20). Besides, ischemia-reperfusion and massive transfusion during thoracic surgery both contribute to the accumulation of leukocytes, ROS and cytokines within the pulmonary microvasculature and signify the degradation of eGCX and injury of endothelium. Fluid overload increases the shear stress which may directly scratch the eGCX layer. It also stimulates the release of atrial natriuretic peptide (ANP) which is associated with increased degradation of eGCX, although the underlying mechanism remains unclear (21).

### 2.3 Lung transplantation

Lung transplantation is the last therapeutic option for end-stage respiratory diseases. Although the 1- and 5-year survival rates post lung transplantation have increased substantially over the past decades, ischemia–reperfusion injury (IRI) and immune rejection remain the severe postoperative complications leading to treatment failure (22). Pulmonary IRI after lung transplantation is the main reason for primary graft dysfunction (PGD), which is a major cause of mortality and morbidity in the postoperative period (23). IRI results in noncardiogenic pulmonary edema and diffuse alveolar damage, and later bronchiolitis obliterans syndrome and graft failure (24). Degradation of the eGCX may be the earliest event when the IRI occurs within the pulmonary microvasculature, which further impairs the local microcirculation *via* vasoconstriction, leukocyte adherence, and activation of the immune response (25, 26). eGCX degradation during posttransplant IRI can be evidenced by elevated plasma levels of its constituents such as heparan sulfate and syndecan-1, which have been proposed as biomarkers of endothelial integrity. By contrast, the decrease of these compounds in the circulation may predict graft acceptability or successful protective interventions against IRI according to the study of Sladden et al (25). Luckily, pulmonary eGCX degradation induced by IRI has been reported to be restored by some anesthetics such as lidocaine and sevoflurane, which indicates a modality ameliorating transplantation associated IRI (27). Degradation of eGCX may also exacerbate immune rejection. While the mechanisms remain unclear, recent studies indicate that eGCX degradation constituents, in particular hyaluronan, are likely involved in rejection (28). The latest finding that protecting the eGCX in vascular allografts attenuates the acute and chronic rejection after transplantation underlines the protective role of an integral eGCX layer in organ transplantation (29). Another study by Coulson-Thomas, et al. reported that umbilical cord mesenchymal stem cells (UMSCs) can inhibit the adhesion and invasion of inflammatory cells and the polarization of M1 macrophages by synthesizing a rich extracellular glycocalyx composed of the chondroitin sulfate-proteoglycan versican bound to a heavy chain (HC)-modified hyaluronan (HA) matrix (HC-HA) (30). Considering these components also exist in eGCX, this finding may reveal a potential mechanism by which eGCX prevents immune rejection.

### 2.4 Pulmonary virus infection

The respiratory tract is in direct contact with the outside environment. Therefore, the lung is often attacked by a wide variety of inhaled pathogens including viruses. It is not uncommon for pulmonary infection to develop into a life-threatening systemic infection or even multiple organ dysfunction syndrome (MODS) due to excessive leukocyte recruitment and activation, and an overzealous inflammatory response. Histologically, the alveolar epithelium and the adjacent pulmonary microvasculature constitute the gas-exchange surface, or the air–blood barrier. The pulmonary eGCX coating on the surface of endothelium is an important composition in the air-blood barrier. Pulmonary eGCX is also frequently damaged following endothelial dysfunction caused by respiratory viral infections, including coronavirus-2019 (COVID-19) infection (31, 32). It has been recognized that pro-inflammatory cells and soluble factors play central roles in this process. During viral infections, resident macrophages in the lung, mainly alveolar macrophages, rapidly respond to inhaled viruses *via* the highly coordinated recruitment of specific innate and adaptive leukocytes from circulation and trigger heavy inflammatory responses. This process can be exemplified by the infection of COVID-19. Like in other pulmonary infections, leukocyte recruitment to the lungs with COVID-19 infection is orchestrated by specific trafficking inflammatory factors (33). When uncontrolled and excessive it can result in various pathological complications inside or outside the lungs (34, 35). There can be collateral damage in this process such as the disruption of pulmonary eGCX layer, which may signify inflammation and vascular damage resulting in vascular leakage and thrombosis. The endotheliopathy caused by eGCX damage during COVID-19 infection does not meet the criteria of systemic inflammatory response syndrome (SIRS), although pathologically it is similar to that of shock. The resulting eGCX associated endothelial disorders have been newly named systemic inflammation-reactive microvascular endotheliosis (SIRME) in some studies. SIRME is manifested by the simultaneous presence of active inflammation (fever, high levels of C-reactive protein and proinflammatory cytokines), endothelial damage with strong thrombogenic tendencies (high D-dimer and fibrinogen degradation products (FDP)) increased vascular permeability, and organ damage (increased respiratory rate, high levels of lactate dehydrogenase and transaminases, and elevated myocardial deviation enzymes). The emergence of SIRME can then turn back to strengthen the degradation of eGCX and exacerbate the vascular disorders, giving rise to a vicious cycle. Moreover, high blood levels of eGCX fragments, or vigorous infiltrative shadows in both lungs indicate progressive SIRME, a high-risk condition for progression to disseminated intravascular coagulation (DIC) or acute respiratory distress syndrome (ARDS) with poor prognosis (31, 36).

### 2.5 Acute respiratory distress syndrome

Acute respiratory distress syndrome (ARDS) is a syndrome of acute onset non-cardiogenic respiratory failure which often leads to severe oxygenation impairment. The capillary endothelium and alveolar epithelium are damaged, with fluid leaking from the vasculature to the alveolar space, leading to pulmonary edema and ARDS. The endothelium becomes inflamed and activated by the adhered leukocytes which drives eGCX degradation while disrupting vascular integrity and increasing permeability, resulting in the leakage of plasma and large amounts of proinflammatory factors across the air-blood barrier into the alveolus (37, 38). The pulmonary eGCX maintains the vascular integrity *via* several pathways. First, it can serve as a passive barrier to at least transiently preclude the direct adhesion of circulating leukocytes to endothelial cells, preventing primary damage and blocking the efflux of proteins and fluid from the pulmonary vasculature (39, 40). Second, the eGCX functions as a mechanotransducer by regulating the contractility of the endothelial cytoskeleton in response to pressure and shear stress within the vascular lumen (41, 42). In addition, pulmonary eGCX may enhance the link of mechanical stimuli with metabolic and inflammatory alterations in the pulmonary microvasculature. The hydrostatic increases within the pulmonary microvasculature contribute to a “proinflammatory” endothelial cell phenotype with increased neutrophil activation and adhesion, a critical step in endothelial injury (43). The release of heparan sulfate following degradation of the eGCX augments neutrophil induced pulmonary injury and may also impact the Na^+^-K^+^ ATPase located on alveolar epithelium, which further disturbs the liquid equilibrium across alveolus and endothelium (13, 43).

### 2.6 Pulmonary arterial hypertension

Pulmonary arterial hypertension (PAH) is defined by an elevated mean pulmonary arterial pressure (mPAP) of more than 25 mmHg. The central initial event of PAH is thought to be vasoconstriction which involves genetic, epigenetic, and environmental mechanisms (44). The mechanical activity of vasoconstriction finally turns to pulmonary vascular remodeling with pulmonary arterial endothelial cell dysfunction and arterial smooth muscle cell proliferation (45). Although no direct evidence supports a role of eGCX in PAH pathogenesis, a recent study showed that the plasma levels of heparin sulfate proteoglycan (HSPG), hyaluronan (HA), and syndecan-1 (SDC-1) were elevated in monocrotaline-induced PAH rats in comparison with control group (46). However, rats that were administered exogenous heparin showed reduced levels of HSPG, HA, and SDC-1. These results indicate that destruction of eGCX was involved in the development of PAH, although the mechanism remains unclear.

### 2.7 Lung cancer

Lung cancer is one of the most prevalent malignant diseases worldwide and has the highest mortality over cancers originating from other tissue types. Metastasis during the initial diagnosis of lung cancer accounts for most cases with a low overall survival rate (47). Long distance metastasis is intimately linked to alterations of vascular permeability. There have been multiple modalities proposed for illustrating the extravasation of circulating malignant cells, including disruption, degradation, down-regulation and phosphorylation of cell junction molecules together with endothelial cell contraction (48). The degradation of eGCX appears to inevitably underlie the weakening endothelial barrier function and enhanced vascular permeability that can mobilize malignant cells (49).

## 3 Potential mechanisms linking eGCX to the pathogenesis of HPS

The pathogenesis of HPS mostly involves pulmonary vascular dilatation in the context of chronic hepatic diseases (50). HPS is always accompanied by global inflammation and hemodynamic disturbance (51). Inflammation is one of the most important contributors to eGCX damage as well as to HPS occurrence. As eGCX is also a sensitive responder to hemodynamic change, in combination with the characteristic change of pulmonary vascular tone during HPS, it seems likely that the eGCX is intimately involved in HPS pathogenesis.

### 3.1 The pathogenesis of HPS

**Figure 2:**
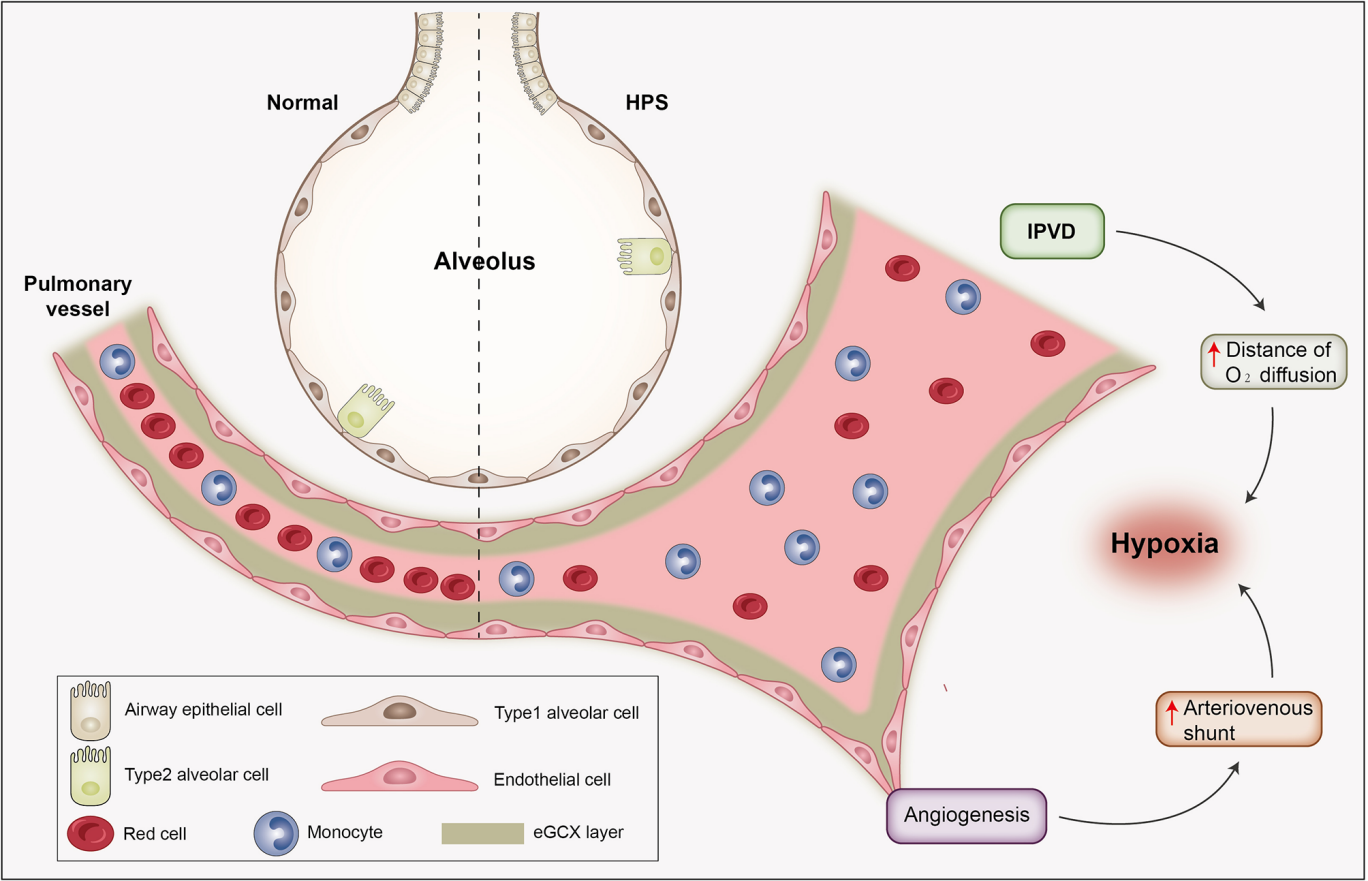
The pathophysiological alterations of HPS. HPS develops with two central pathogenic features—vasodilatation and angiogenesis. Intrapulmonary vascular dilation is the most important pathological process in HPS, resulting in elongated distance of oxygen diffusion and incomplete oxygenation of returned venous blood. Angiogenesis gives rise to intrapulmonary shunt and exacerbates ventilation/perfusion mismatch. These collectively lead to gas exchange abnormality and hypoxia. Abbreviation: IPVD, intrapulmonary vascular dilation.

HPS develops with two central pathogenic features—pulmonary microvascular dilatation and angiogenesis, which collectively lead to gas exchange abnormality and impaired oxygenation (**Figure 2**). Intrapulmonary vascular dilation (IPVD) is the most important alteration in HPS, resulting in the impaired oxygenation of returned venous blood. Insight into the pathogenesis of HPS derives principally from experimental studies using animal models, especially the CBDL rat model (52). The emergence of IPVD is attributed to multiple mechanisms which involve a variety of inflammatory cells, cytokines, growth factors, and hemodynamic parameters (1). Vasodilation is triggered by excessive nitric oxide (NO) release through type B endothelin receptor (ET_B_) signaling driven by endothelial nitric oxide synthase (eNOS) activation and inducible nitric oxide synthase (iNOS) production in marginal monocytes within the pulmonary vasculature (52). Additionally, carbon monoxide production in monocytes mediated by heme oxygenase 1 (HMOX1) is also involved. Moreover, monocytes that have adhered to the pulmonary endothelium produce angiogenic growth factors such as vascular endothelial growth factor (VEGF) leading to angiogenesis by activating angiogenic signals including Akt and ERK in endothelial cells. Finally, angiogenesis fosters an arteriovenous shunt, which when superimposed on IPVD dramatically exacerbates hypoxemia (53, 54).

### 3.2 The degradation-reconstitution balance of eGCX

The eGCX covering on the pulmonary endothelium is at the frontline against hemodynamic and immune disturbances, particularly under conditions of chronic liver disease. The eGCX is degraded *via* inflammatory mechanisms which trigger the production and activation of metalloproteinases, heparanases, and hyaluronidases (55). In patients with acute diseases such as ischemia-reperfusion injury, hypoxia, and sepsis, high concentrations of fragmented eGCX, which include syndecan-1, syndecan-4, hyaluronic acid, and heparan sulfate, can be detected in the circulation (56–58). The damaged eGCX denudes the surface of vascular endothelial cells, facilitating excessive vascular permeability and leakage, and contributing to further pathological deterioration by causing interstitial edema (59, 60). More importantly, the degradation of eGCX may be balanced by a dynamic reconstitution *via* the synthase exostosin (EXT), which warrants a relatively stable endothelial barrier despite the appearance of IPVD, and prevents extravasation of leukocytes and leakage of fluid and plasma proteins into the interstitial space (61–65). This may explain in part why HPS is not frequently complicated with pulmonary edema at an early stage (66).

### 3.3 The pulmonary eGCX and IPVD

The pulmonary circulation carries deoxygenated blood from the systemic veins through the pulmonary arteries to be oxygenated in the capillaries that line the walls of the pulmonary alveoli. Normally, the pulmonary circulation is of low driving pressure and flow velocity, and maintains a low vascular resistance and low fluid shear stress. In fact, the pulmonary circulation is often considered to be a quasi-static system in both experimental and computational studies so as to match the ventilation for gas exchange and oxygenation (67, 68). Accordingly, the pulmonary eGCX is thinner compared with that in other organs because of a lower rate of glycosaminoglycan synthesis (2, 12, 25). The pulmonary eGCX is also more sensitive to mechanical signals although within an environment of low shear stress in pulmonary circulation (42, 69, 70).

#### 3.3.1 eGCX and pulmonary vascular tone

The disorder of blood flow exerts mechanical tangential forces to the endothelial surface such as shear stress. The mechanical signal of blood flow changes sensed by eGCX subsequently lead to the production of NO, a major regulator of vascular tone (71). In the setting of HPS, the regulation of pulmonary vascular tone appears to be more complicated. Liver diseases are often accompanied by retention of fluid and sodium thanks to elevated aldosterone level, which, in combination with the potential portal-systemic shunt, may contribute to a hyperdynamic state of systemic circulation. It has been show that hyperdynamic circulation leads to damage of eGCX in peripheral vessels (72). However, the pulmonary circulation is relatively tolerant to hemodynamic changes due to its low resistance. This may be protective to pulmonary eGCX, the preservation of which may prevent subsequent insults to the endothelium integrity and reduce the frequency interstitial edema (73, 74). Contrarily, the pulmonary eGCX under this condition may exacerbate IPVD by sensing the increased shear stress and elevating NO release, although this remains to be specifically elucidated in human studies.

With regard to the hypoxia caused by IPVD, a more complicated response for the pulmonary vasculature may exist as well. In those without HPS the pulmonary arteries constrict under hypoxic stimuli which is in contrast to systemic blood vessels that typically dilate in response to hypoxia. This is termed hypoxic pulmonary vasoconstriction (HPV). The mechanisms of HPV involve the release of endothelial derived substances including endothelins, superoxide anions and thromboxane A2, and also by a decrease in NO bioavailability that leads to vascular smooth muscle cell contraction (75). The HPV response ensures that blood flow in locally hypoxic alveoli can be reduced in order to divert the cardiac output to better oxygenated regions, which precludes a further deterioration of the ventilation/perfusion mismatch. However, during HPS, hypoxia seems to be ineffective in driving HPV. The reason may partly be ascribed to the exuberant mechanisms causing IPVD. It has been well known that hypoxia is sufficient to induce degradation of eGCX in pathophysiological conditions (76). Surprisingly, the heparan sulfate proteoglycan deficiency during eGCX degradation is reported to up-regulate the intracellular production of NO, which may alleviate the vascular contraction induced by hypoxia (77, 78). During HPS, the upregulated fibroblast growth factor (FGF) signaling promotes reconstitution of eGCX by enhancing the activity of the synthatase EXT1, which may give rise to the degradation-reconstitution balance of eGCX as mentioned above and offset the direct vasoconstrictive effect of hypoxia. In addition, the paucity of smooth muscle cells in pulmonary microvessels may decrease the effect of hypoxia even though a long term hypoxic environment cultivates vascular remodeling (79, 80).

#### 3.3.2 eGCX and monocyte adhesion

HPS patients are usually under a state of chronic inflammation, which may disrupt the continuity of the eGCX layer and render the pulmonary endothelium more likely to be adhered and affected by circulating leukocytes including monocytes (53). With the advancement of liver disease, there will be a significant elevation of pathogen and damage-associated molecular patterns (PAMPs & DAMPs) and pro-inflammatory mediators (81). Chemokines including MCP1, regulated on activation normal T-cell expressed and secreted (RANTES), and macrophage inflammatory peptides 1α and β (MIP-1α and MIP-1β, respectively) can interact with the side chain components of eGCX to form the so called “chemokine-cloud,” a local concentration of chemokines within the eGCX layer which may facilitate leukocyte adhesion (82). In addition, the pro-inflammatory mediators also include various enzymes such as heparinase, hyaluronidase, matrix metalloproteinases (MMPs) and a disintegrin and metalloproteinases (ADAMs). These enzymes degrade eGCX and contribute to a discrete bareness of the endothelium while exposing receptors on the membrane of endothelial cells, including multiple types of pattern recognition receptors (PRRs), growth factor receptors, cytokine/chemokine receptors and adhesion molecules (55, 58, 83). The cytoskeletal fibers within the endothelium also rearrange in order to orchestrate cell adhesion and focal contact formation (84–86).

An integral layer of eGCX restrains the interaction between the endothelium and circulating white blood cells by preventing them from approaching, while damaged eGCX may facilitate leukocyte attachment. The protective role of eGCX is attributed to the constituents of heparin sulfate proteoglycans (HSPGs) and endomucin (EMCN). Upon inflammatory stimulation, the glycans are shed from the endothelial cell surface, which results in slower rolling and adhesion of leukocytes to the endothelium. Similarly, breakdown of the eGCX increases platelet–vessel wall interactions (36, 87). Monocytes expressing iNOS are the major effector cells driving the pathogenesis of HPS through the modulation of NO (50). The interaction of monocytes with endothelial cells is also on the basis of eGCX damage. The products of eGCX degradation including heparan sulfate and chondroitin sulfate may enhance the chemotactic migration of monocytes, although glypican 1 mounted on the membrane of endothelial cells is reported to inhibit monocyte adhesion (88–90). Hence, the destruction of eGCX may be precisely limited to the area where monocytes are recruited most intensively (91, 92). These recruited monocytes can release enzymes to further degrade the eGCX layer (93). The simultaneous exposure of adhesion molecules and receptors lays the foundation for monocyte-endothelium interactions and IPVD (94, 95), and subsequent regulation of the aforementioned eGCX degradation-reconstitution equilibrium (61, 62). This mode makes it possible for IPVD to occur without obvious dysregulation of vascular permeability since the adhered monocytes could be restrained by glypican 1 on the endothelium. However, it remains to be demonstrated in both experimentally and in clinical studies.

#### 3.3.3 eGCX and NO production

**Figure 3:**
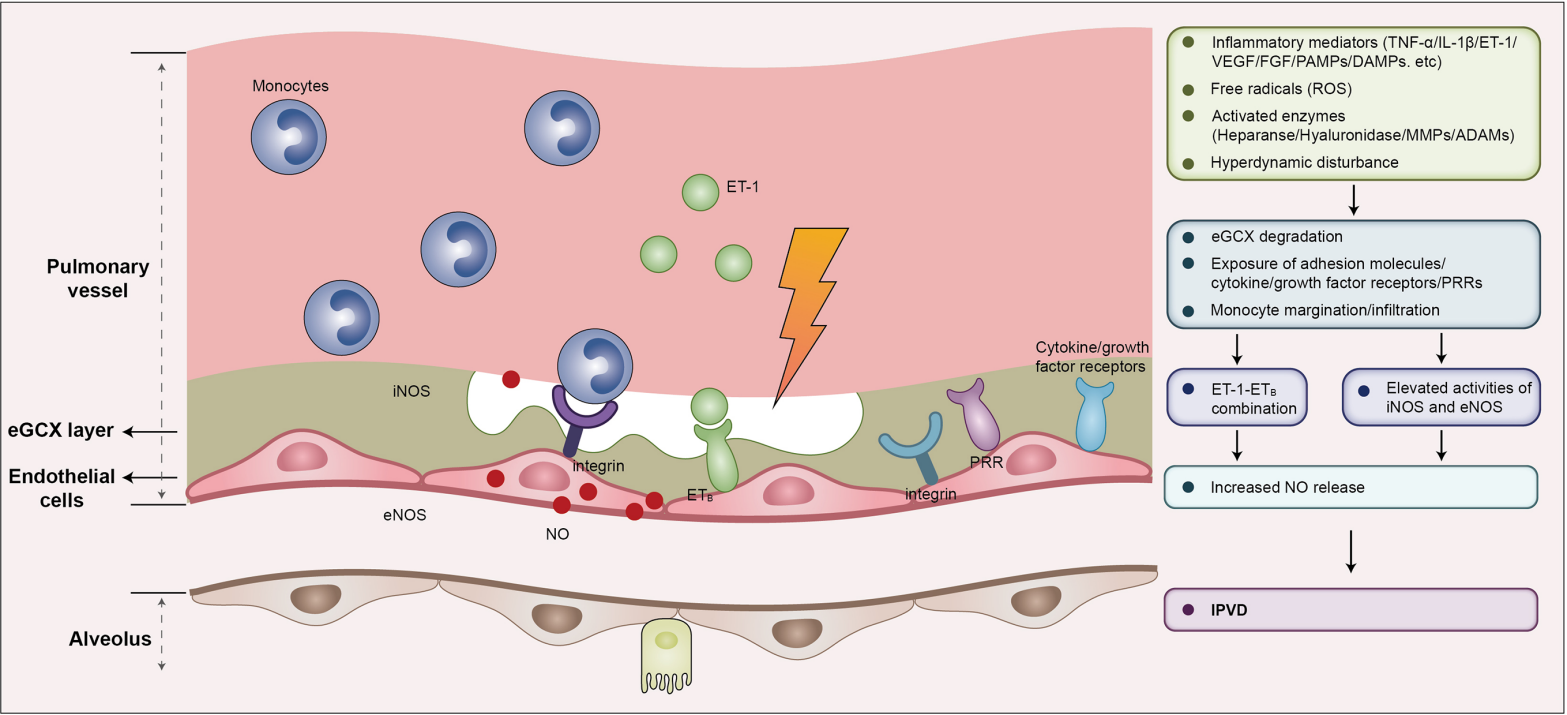
eGCX may play an important role in HPS associated IPVD. During HPS, eGCX degradation exposes adhesion molecules, cytokine/growth factor receptors, and pattern recognition receptors that facilitate the processes of monocyte migration and infiltration, and the interaction of monocytes and endothelial cells, resulting in increased expression of iNOS and eNOS, and the consequent release of NO, the major contributor of IPVD. Abbreviations: eGCX, endothelial glycocalyx; IPVD, intrapulmonary vascular dilation; iNOS, inducible nitric oxide synthase; eNOS, endothelial nitric oxide synthase; NO, nitric oxide; ET-1, endothelin-1; ETB, type B endothelin receptor; VEGF, vascular endothelial growth factor; FGF, fibroblast growth factor; ROS, reactive oxygen species; TNF-α, tumor necrosis factor α; IL-1β, interleukin-1β; PAMPs, pathogen associated molecular patterns; DAMPs, damage associated molecular patterns; PRRs, pattern recognition receptors; MMPs, matrix metalloproteinases; ADAMs, a disintegrin and metalloproteinases.

The eGCX plays a pivotal role in vascular mechanotransduction in response to hemodynamic fluctuation. Blood flow exerts tangential shear stress on the endothelium, which is sensed by the eGCX and triggers the reorganization of the actin cytoskeleton and activation eNOS (96, 97). The induction of eNOS is related to glypicans, a core constituent of eGCX embedded in the endothelial membrane (98, 99). The eGCX also responds to mechanical force by inducing a variety of sensors expressed by endothelial cells such as G–protein–coupled receptors, integrins and adhesion molecules. Circulating pro-inflammatory mediators may bind to these molecules, resulting in a disturbance of cellular cytoskeleton kinetics and increased expression of iNOS. eNOS and iNOS both facilitate the release of NO and induce a widespread luminal dilation of the pulmonary microvasculature (52) (**Figure 3**). Additionally, the syndecan-1 and syndecan-4 contained in eGCX interact with cytoskeleton proteins to orchestrate leukocyte contact and adhereance to the endothelium. This serves to enhance NO expression by releasing more cytokines and thus driving a vicious pathogenic feed-forward circle (100–102). The release of heparan sulfate and hyaluronan in eGCX impairs NO production and reduces vasodilation as well (103). In the context of HPS, the hemodynamic discrepancy and elevated pro-inflammatory mediators may damage the pulmonary eGCX and enhance endothelin 1/endothelin receptor B (ET-1/ET_B_) signal-induced expression of eNOS and NO (104, 105).

### 3.4 The pulmonary eGCX and angiogenesis

Angiogenesis is characterized by the sprouting of neovasculature from pre-existing vessels, which comprises the processes of endothelial cell proliferation, migration, and tube formation (106). Angiogenesis takes place normally in development and pathologically in response to inflammatory and (or) ischemic/hypoxic stimuli (107). During HPS, angiogenesis occurs vigorously within the pulmonary vascular network, leading to arteriovenous shunt and exacerbating the hypoxemia caused by IPVD associated ventilation/perfusion mismatch (108–110). The eGCX components have been reported to play important roles in angiogenesis in both homeostatic and pathological circumstances (111). However, whether and how the pulmonary eGCX contributes to angiogenesis during HPS remains unclear.

#### 3.4.1 eGCX and angiogenic signals

Under homeostatic conditions, the eGCX components mediate angiogenesis *via* a variety of mechanisms. Heparin sulfate can bind to circulating factors such as vascular endothelial growth factor (VEGF) and fibroblast growth factor (FGF) and initiate angiogenic signaling in endothelial cells by regulating their bioavailability, local concentrations, and stability (112–115). Syndecans play distinct roles in angiogenesis either by modulating VEGFR2 internalization, or by binding to VEGF as a co-receptor or inhibitory receptor (116–119). EMCN promotes angiogenesis *via* VEGFR2 internalization as well (120, 121). The function of hyaluronan (HA) in angiogenesis appears to be more complicated. HA exerts an anti-angiogenic effect during homeostasis, whereas after degradation the products can induce angiogenesis by activating HA receptors CD44 and CD168 (122–124).

Angiogenesis in HPS appears to result from pulmonary inflammation, with accumulated monocytes producing angiogenic factors and vasoactive mediators directly affecting pulmonary endothelium (125). Pulmonary endothelial cells can also be influenced by autocrine signals (53). In the setting of HPS, damage to the eGCX layer leads to the exposure of the receptors of angiogenic factors. The factors that are reported to act in HPS include VEGF-A, platelet growth factor (PDGF), and placental growth factor (PlGF) (110, 126). VEGF-A/VEGFR2 has been demonstrated to be the major angiogenic signal in HPS associated angiogenesis (127, 128). Both intravascular monocytes and pulmonary endothelial cells produce VEGF-A. The VEGF molecule contains heparan-binding domains and can be activated when combined with heparin sulfate irrespective of proteoglycan binding within the eGCX layer or free in the plasma (112, 129). At the site of angiogenesis, the disrupted eGCX layer may provide the location for monocyte-endothelial cell interaction to accommodate angiogenic signal transduction and migration, and the subsequent proliferation of endothelial cells (130, 131). During this process, the EMCN within the eGCX layer facilitates VEGFR2 internalization and downstream signaling as previously mentioned. Some systemically elevated factors may promote VEGFR2 internalization as well during HPS, such as oncostatin M (OSM) and Galectin-1/3 (132, 133). However, since most of these results are based on experimental HPS models, whether identical mechanisms exist in humans remains to be determined.

#### 3.4.2 eGCX synthesis during angiogenesis

**Figure 4:**
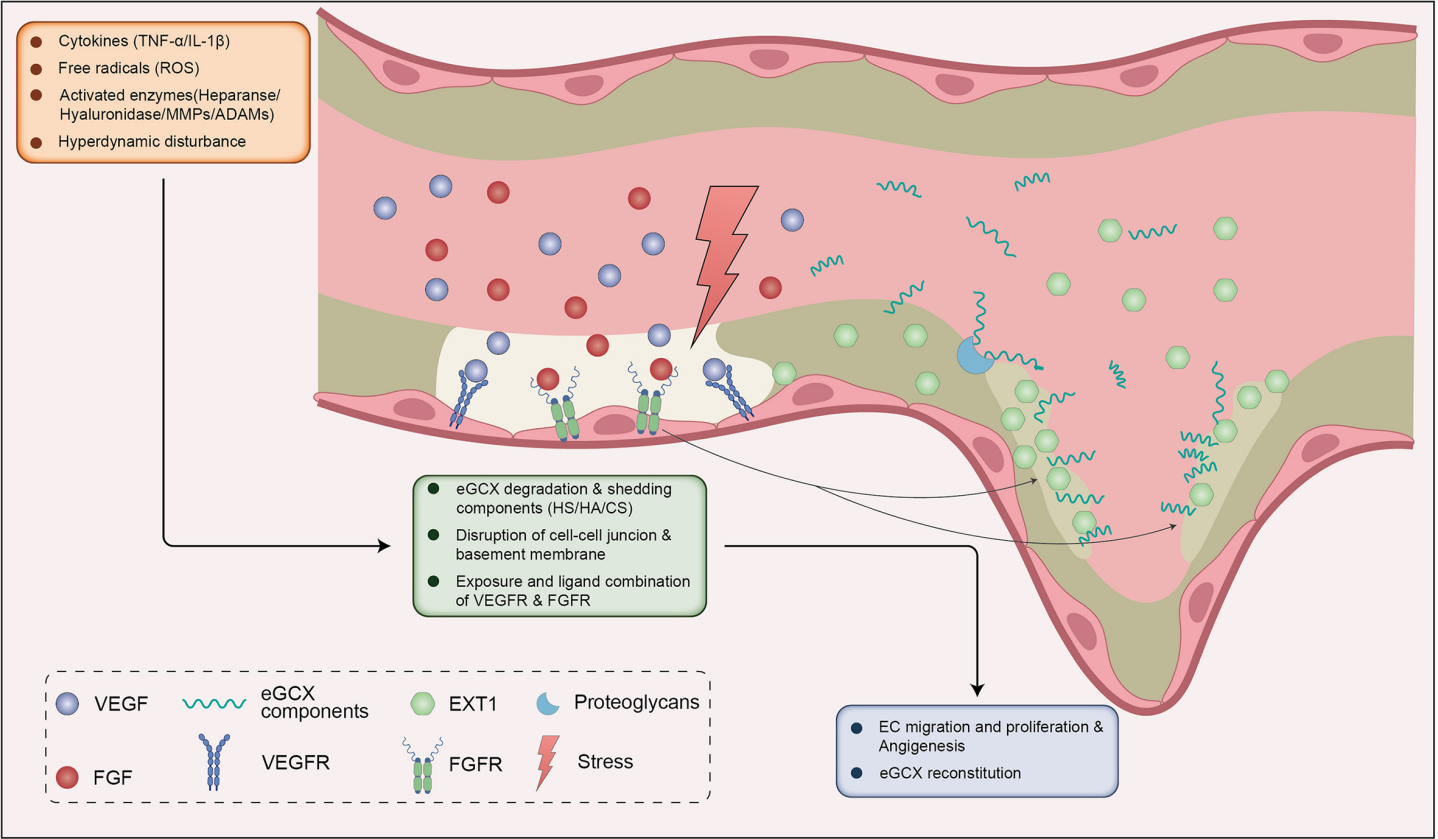
eGCX may be involved in HPS associated angiogenesis. During HPS, eGCX degradation also leads to exposure of VEGFR and FGFR, ensuing the combination of VEGF and FGF respectively. VEGF-VEGFR and FGF-FGFR signals promote the migration and proliferation of ECs. The disruption of eGCX continuity promotes the destruction of cell-cell junctions and the basement membrane, which together with ECs proliferation and migration facilitates the sprout of neovessel. The FGF-FGFR signal activates the downstream enzyme EXT1 to reconstitute the eGCX layer within the injured site and neovessels (in the dashed line frame). The shedded eGCX components are absorbed by the proteoglycans on the EC membrane and enhance the reconstitution of eGCX. Abbreviations: eGCX, endothelial glycocalyx; HS, heparin sulfate; HA, hyaluronic acid; CS, chondroitin sulfate; VEGFR, vascular endothelial growth factor receptor; FGFR, fibroblast growth factor receptor; ROS, reactive oxygen species; TNF-α, tumor necrosis factor α; IL-1β, interleukin-1β; MMPs, matrix metalloproteinases; ADAMs, a disintegrin and metalloproteinases; EXT1, exostosin 1; EC, endothelial cells.

Following the proliferation of endothelial cells, the composition of the eGCX layer is important for the formation of normal vascular morphology and functions in the process of tube formation (114, 134, 135). Synthesis of the eGCX is initiated by the enzyme EXTL1-3 with the side chain elongation mediated by EXT1-2 (136). It has been proposed that there is a dynamic equilibrium between the shedding of eGCX components under pathologic conditions, the adsorption of the components from circulating blood, and synthesis of eGCX, which indicates a tendency to maintain an intact eGCX layer within afflicted vessels (137, 138). During HPS, both inflammation and hemodynamic disturbances can stimulate the degradation of eGCX. Damage to the eGCX layer exposes the endothelium to inflammatory mediators and enzymes which then disrupt endothelial cell-cell junctions and the basement membrane, and facilitates the budding of neovessels (139, 140). The local degradation of eGCX increases shedding of heparan sulfate, hyaluronan and chondroitin sulfate. These components on the one hand are adsorbed by the bared or the newborn endothelium (137). On the other hand, they may activate the eGCX synthetic signals of FGF/FGFR1/EXT1, VEGF/VEGFR2, S1P/PI3K and Ang1/Tie2 (61, 62, 141, 142). Hence, the eGCX layer is probably formed on newborn endothelium with the help of shedded components that originated from the location of the vessel sprouts (137, 138) (**Figure 4**).

### 3.5 The potential involvement of eGCX in HPS pathogenesis

**Figure 5:**
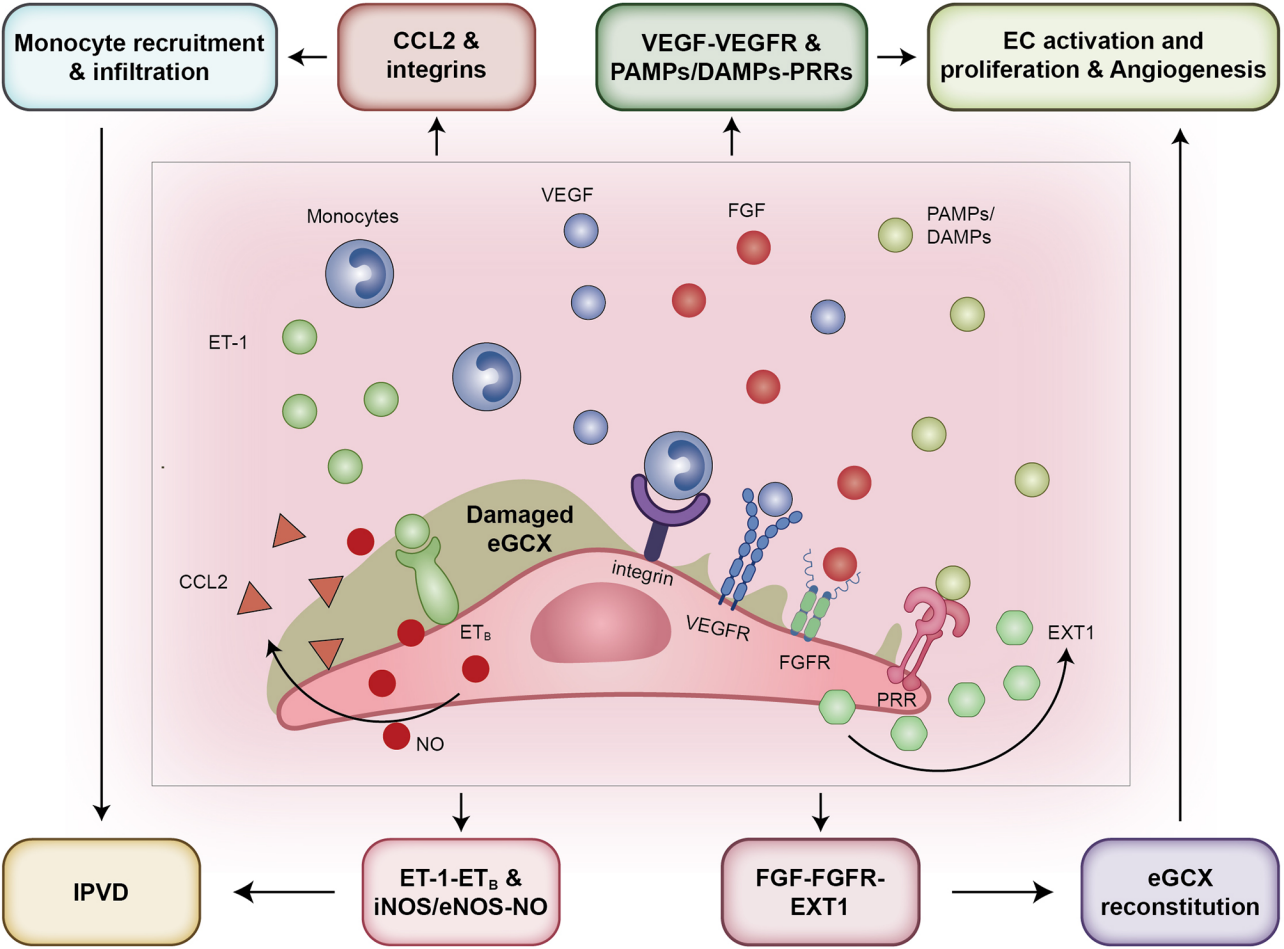
The pathways that eGCX adopts to participate in HPS pathogenesis. From the left to the right: First, eGCX degradation promotes the contact of monocytes with the endothelium by exposing adhesion molecules such as integrins and selectins, contributing to elevated expression of iNOS and eNOS and subsequently NO release. Second, the exposure of receptor ET_B_ when bound by ligand ET-1 increased the release of NO as well. Third, the VEGF-VEGFR and FGF-FGFR signals activated after eGCX degradation promote the migration and proliferation of ECs, facilitating the sprout of neovessels. Fourth, the FGF-FGFR signal activates the downstream enzyme EXT1 to reconstitute the eGCX layer within the injured site and neovessels. Abbreviations: eGCX, endothelial glycocalyx; IPVD, intrapulmonary vascular dilation; CCL2, C-C motif chemokine ligand 2; ET-1, endothelin-1; ETB, type B endothelin receptor; VEGFR, vascular endothelial growth factor receptor; FGFR, fibroblast growth factor receptor; PAMPs, pathogen associated molecular patterns; DAMPs, damage associated molecular patterns; PRRs, pattern recognition receptors; PRRs, pattern recognition receptors; NO, nitric oxide; iNOS, inducible nitric oxide synthase; eNOS, endothelial nitric oxide synthase; EXT1, exostosin 1; EC, endothelial cells.

The current evidence supporting eGCX alterations in pulmonary disorders may be described in a five-step schematic which outlines such alterations in the context of HPS pathogenesis. First, global inflammation and hyperdynamic disturbance stimulate the local degradation of pulmonary eGCX during HPS. The damaged eGCX layer exposes endothelial adhesion molecules (integrins, selectins, etc), cytokine and growth factor and pattern recognition receptors, and the endothelium itself to immune cells, inflammatory mediators and angiogenic factors. Second, recruited monocytes adhere to the endothelium, elevate the expressions of iNOS and eNOS, and subsequently facilitate the production of NO, which are the most important vasodilator contributing to IPVD. The chemokines released by activated endothelial cells further recruit more monocytes and dramatically expand the vasodilative effect, leading to exacerbated IPVD and hypoxia in HPS. Third, increases in circulating FGF promotes reconstitution of the eGCX layer by enhancing the activity of the synthase EXT1, which gives rise to the degradation-reconstitution balance of eGCX and recovers the functions of eGCX in mechanotransduction and regulation of vascular tone. This takes place in a hyperdynamic state during HPS, and at least partially offsets the direct vasoconstrictive effect caused by hypoxia. Fourth, the inflammatory mediators, especially ROS and MMPs, disrupt endothelial cell-cell junctions and the basement membrane in exposed endothelial sites, and facilitate the bud of neovessels. Last but not the least, the eGCX layer is probably reformed on the newborn endothelium with the help of the shedded components that originate from where the eGCX degrades and vessels sprout. These components are adsorbed by the endothelium and activate the eGCX synthetic signals such as FGF/FGFR1-EXT1, VEGF/VEGFR2 and Ang1/Tie2, leading to (re)construction of the eGCX layer in parental and daughter vessels (**Figure 5**).

## 4 Discussion

Therapeutic strategies targeting the eGCX has been put forward in a variety of reports and include fluid and electrolyte management, blood glucose control, administration of glucocorticoid, supplementation of the eGCX components, inhibition of the degrading enzymes, application of glycocalyx-mimetic biomaterials or nanomaterials, and others (143–145). These therapeutic modalities all aim to prevent excess damage, reconstitute the integrity, or even replace the destructed eGCX layer. Similar strategies have been widely investigated in a variety of studies on pulmonary disorders including acute respiratory distress syndrome (ARDS), sepsis associated lung injury, COVID-19 infection, and lung transplantation (**Table 1**). Amongst these therapeutic interventions, anti-inflammatory effects with decreased leukocyte adhesion and cytokine secretion are commonly described, reflecting the primary importance of inflammation in causing the discrepancy of pulmonary eGCX. Besides, these studies also reveal that ROS and a series of sheddases such as heparanase, hyluronase, and MMPs are potential targets against pulmonary eGCX degradation. Much impressive results are from the studies of FGF, protectin conjugates in tissue regeneration 1 (PCTR1), maresin conjugates in tissue regeneration 1 (MCTR1), Colivelin and Fucoidan, which show the reconstitution effect on pulmonary eGCX *via* the signals of FGFR1/EXT-1, SIRT1/NF-κB p65/EXT-1, STAT3/AMPK, ANG2 respectively (61, 63, 152–154). Clinically, the aim of reconstituting eGCX should be emphasized in combination with the treatments of anti-inflammation and inhibition of ROS and sheddases provided that it is likely to preclude or delay the occurrence of vascular leakage and the consequent lung edema (136, 158). However, it should be taken into consideration given that the involving signals such as FGFR1 activation, NF-κB p65 and STAT3 inhibition, and ANG2 downregulation may laterally contribute to exacerbated organ fibrosis, compromised immunity against pathogens and angiogenesis-associated intrapulmonary shunt (125, 159–161), which may not only complicate lung injury and hypoxia but also lead to other organ dysfunctions. Besides, the means of inhibiting hyluronase and MMPs may also cause or worsen organ matrix deposition, in particular when comorbid with liver fibrosis (162). Inhibition of heparanase may interfere the cogulation system and increase the risk of bleeding (163). Further studies are needed to find an optimal modality which balance the protection and restoration of pulmonary eGCX and the adverse effects.

**Table 1:** Therapeutic interventions to protect or restore the pulmonary eGCX.

Therapeutic intervention	Study type	Effect	Mechanism	Reference
Albumin	*In vitro* study of the mechanical properties of bovine lung endothelial cells	Increasing the thickness and reducing the stiffness of eGCX	Likely protecting the eGCX from hyaluronidase and interacting with hyluronon	(42)
Plasma	Rat model of hemorrhagic shock	Restoring pulmonary eGCX	Restoring cell surface syndecan-1 by mobilizing an intracellular pool of preformed syndecan-1 Stimulating endothelial cell syndecan-1 transcription	(146)
Heparin	*In vitro* study of the effects of plasma from COVID-19 patients on human umbilical vein endothelial cells (HUVECs)	Preventing eGCX degradation	Inhibiting heparanase	(147)
Antithrombin	Mice model of LPS induced ARDS	Attenuating pulmonary eGCX damage	Decreasing neutrophil infiltration and cytokine secretion	(39)
Thrombomodulin	Mice model of Streptococcus pneumoniae-induced sepsis	Attenuating pulmonary eGCX damage	Diminishing systemic inflammation and hypercytokinemia	(148)
Dexamethasone	COVID-19 patients	Decreasing pulmonary eGCX degradation and ameliorating endothelial injury	Decreasing inflammation and leucocytes adhesion	(149)
Sevoflurane	Pig model of *in-vivo* lung autotransplant	Preserving pulmonary eGCX layer	Decreasing leukocyte recruitment and adhesion Protecting pulmonary eGCX from ischemia reperfusion injury	(150)
Doxycycline	Mice model of intratracheal LPS-induced lung injury	Reducing pulmonary eGCX degradation	Inhibiting heparanase and MMP	(151)
Ulinastatin	Mice model of LPS-induced ARDS.	Attenuating pulmonary eGCX damage	Reducing the active form of heparanase expression and inhibiting heparanase activity.	(87)
Fibroblast growth factor (FGF)	Patients with nonpulmonary sepsis or pneumonia Mice model of sepsis induced by cecal ligation and puncture (CLP)	Promoting pulmonary eGCX reconstitution	Inducing of the heparan sulfate (HS) biosynthetic enzyme exostosin (EXT)-1 *via* FGFR1 signaling	(61)
Protectin conjugates in tissue regeneration 1 (PCTR1)	Mice model of LPS-induced pulmonary eGCX loss	Inhibiting pulmonary eGCX degradation and promoting reconstitution	Inducing SIRT1 expression and reducing NF-κB p65 phosphorylation	(63)
Maresin conjugates in tissue regeneration 1 (MCTR1)	Mice model of LPS-induced sepsis	Attenuating pulmonary eGCX injury	Decreasing inflammatory cytokines Downregulating heparanase expression Upregulating SIRT1 expression and decreasing NF-κB p65 phosphorylation	(152)
Colivelin	Mice model of sepsis induced by cecal ligation and puncture (CLP)	Ameliorating pulmonary eGCX degradation and endothelial injury	Inhibiting the activation of STAT3 Increasing activation of AMPK	(153)
Fucoidan	COVID-19 patients *In vitro* study of patient sera on primary lung microvascular endothelial cell (HPMEC)	Restoring eGCX layer	Reducing endothelial activation through inactivation of NF-κB signaling pathway and downstream ICAM1 expression Decreasing ANG2 expression Reducing endothelial cell surface tissue factor (TF)	(154)
Berberine	Mice model of LPS-induced ARDS.	Alleviating pulmonary eGCX degradation and promoting restoration	Inhibiting ROS, heparanase, and MMP-9 Decreasing the production of pro-inflammatory cytokines Inhibiting NF-κB signaling pathway activation	(155)
Crocin	Mice model of LPS-induced ARDS.	Alleviating pulmonary eGCX damage and degradation	Inhibiting the expressions of cathepsin L, heparanase, and MMP-9 Reducing neutrophil adhesion or infiltration Inhibiting HMGB-1, NF-κB and MAPK signaling pathways	(156)
Fraxin	Mice model of LPS-induced ARDS.	Protecting pulmonary eGCX from degradation and reducing vascular permeability	Inhibiting the production of inflammatory factors and the activation of NF-κB and MAPK signaling pathways Inhibiting reactive oxygen species (ROS) increase and lipid peroxidation Increasing the superoxide dismutase (SOD) activity to avoid oxidative damage.	(157)

eGCX, endothelial glycocalyx; LPS, lipopolysaccharide; ARDS, acute respiratory distress syndrome; COVID-19, Corona virus disease-19; MMP, matrix metalloproteinase; SIRT1, sirtuin 1; NF-κB, nuclear factor kappa-B; AMPK, AMP-activated protein kinase; MAPK, mitogen-activated protein kinase; STAT3, signal transducer and activator of transcription 3; HMGB1, high mobility group box 1; ICAM 1, intercellular cell adhesion molecule 1; ANG2, angiopoietin 2;

With respect to HPS treatment, it seems likely that protection of the eGCX layer from degradation is necessary. Theoretically, the eGCX can isolate the endothelium from circulating monocytes, inflammatory mediators, and enzymes that disrupt vascular continuity, and will therefore protect the pulmonary vasculature from IPVD, vascular leakage, and interstitial and alveolar edema. Furthermore, a continuous eGCX layer can prevent proangiogenic factors from binding to receptors, which can initiate HPS associated angiogenesis and shunt. These effects likely give rise to the improvement of both ventilation/perfusion mismatch and hypoxia.

However, there has been no direct evidence demonstrating a relationship between the eGCX and HPS. Recently, in the preliminary study, we found that the release of heparan sulfate and hyaluronan, two major components of eGCX side chains, increased significantly in the lungs of HPS rats. Despite this, a dramatic destruction of eGCX layer in pulmonary vessels histologically during the early stage of HPS was not observed (data unpublished). Nevertheless, in the highly proinflammatory environment accompanying liver cirrhosis, the pulmonary eGCX would inevitably be affected, especially considering the pleiotropic roles of eGCX in regulating vascular behaviors and in modulating monocyte recruitment, and the crucial pathophysiological function of monocytes in HPS pathogenesis (55, 82, 136, 152). Given that the eGCX layer is constantly undergoing degradation-reconstitution regardless of physiological or pathophysiological conditions (153), we prefer to speculate that the pulmonary eGCX may adopt a degradation-reconstitution equilibrium mode in the pathogenesis of HPS rather than maintain a persistent uninfluenced state. This type of dynamic alteration would be difficult to detect at the histological level and may explain the paucity of studies detailing the participation of pulmonary eGCX in HPS progression. Therefore, future studies are urgently needed to elucidate the temporal and spatial alterations of pulmonary eGCX structures during HPS.

As the signal factors for eGCX synthesis and the profibrotic factors promoting liver fibrosis, FGF and VEGF have been reported to be elevated in a number of studies on biliary cirrhosis (154, 159, 160). It has been described that the eGCX is able to directly interact with circulating factors *via* the sulfated side chain, mostly heparin sulfate. Binding of these factors to heparin sulfate strengthens their stability and bioavailability by preventing their degradation, and also leads to a local increase of their concentration on the surface of endothelium (111). Disruption in the continuity of eGCX layer by inflammation can expose the receptors of these factors. Theoretically, the combination of FGFR with FGF and VEGFR with VEGF *via* downstream signaling probably enhances the activity of the eGCX synthase EXT as previously mentioned. This pathway may optimally drive the reconstitution of pulmonary eGCX layer, yet will still need to be confirmed in HPS by experimental and clinical studies. From the therapeutic point of view, targeting FGF or VEGF to improving lung disease or ameliorating liver cirrhosis may be an intractable problem in HPS treatment. Simply administering extrinsic FGF or VEGF to improve pulmonary vascular lesions and hypoxia, or administering FGFR or VEGFR antagonists to delay the progression of liver cirrhosis seems much more inappropriate as each may exacerbate the situation of the other. Studies are needed to address the paradox. In addition to FGF and VEGF, the other two factors angiopoietin 1 (Ang1) and Sphingosine-1 (S1P), which upregulate the expression and extravasation of eGCX components (mainly the core protein syndecan) *via* PI3K and Tie2 signals respectively, are also elevated during liver fibrosis (161, 162). Generally, the side chains of eGCX are the first line to confront the proinflammatory disturbance and are thus more susceptible to sheddase. If the core proteins remain intact, the eGCX layer would be easier to reconstitute by EXT. While if the core proteins are badly disrupted, the function of Ang1 and S1P in eGCX reconstitution should be emphasized (2, 136). What it means to treatments of targeting pulmonary eGCX for HPS is still unknown. Besides, there are numerous cytokines, chemokines and complements elevated during liver cirrhosis which are capable of interacting with eGCX (5, 163). Whether and how these factors impact on the degradation-reconstitution process of pulmonary eGCX and the pathogenesis of HPS remains unclear. Further studies are urgently required to find out the answers.

## 5 Conclusions

It has been demonstrated in recent decades that the eGCX plays a crucial role as a microvascular endothelial barrier that maintains microcirculatory homeostasis. The pulmonary eGCX has been shown to modulate the pulmonary circulation and thus participate in a number of pulmonary disorders. Although it is still unknown whether and how the eGCX influences the pathogenesis of HPS, the established functions of eGCX in the inflammatory response, hemodynamic and vascular tone alterations, and angiogenesis may allow us to unmask its roles in HPS-associated IPVD and angiogenesis. Overall, our review primarily unveils the functions of eGCX in the pathogenesis of HPS *in vitro* and *in vivo*, providing a potentially valuable therapeutic target for the treatment of this disorder.

## Author contributions

The review was conceptualized by LL, JJ and SL. LL and CC drafted the manuscript. YL and JL edited and reviewed the manuscript. All authors contributed to the article and approved the submitted version.
